# Supplementary material for: Evaluation of a Semi-Automated Ultrasound Guidance System for Central Vascular Access
Source: Bioengineering (Basel). 2024 Dec 15;11(12):1271. doi: 10.3390/bioengineering11121271 (PMC11673238; doi:10.3390/bioengineering11121271)
Supplement: Supplementary file 1 [file bioengineering-11-01271-s001.zip › bioengineering-3345763-supplementary.pdf]

Supplementary Materials

Supplementary Table S1. Summary of survey responses across all study participants (*n* = 20) for evaluation of the Vu-Path™ device.

Question	Percent Yes Response
**User Experience Related**	
Prior to participating in this study, I was familiar with the use of ultrasound for obtaining vascular access.	**95%**
I have personally used an ultrasound machine in my work.	**90%**
I have personally used ultrasound for obtaining vascular access.	**70%**
**Device Training Related**	
Was the intended use of the device clearly stated as part of the training?	**100%**
Were risks associated with using the device outlined in training?	**95%**
Was the training you received easy to understand?	**100%**
Did you feel adequately trained on the equipment?	**100%**
Was the training time reasonable, ie. not too short or too long?	**100%**
**Device Design Related**	
Aesthetically, did you like how the product looks?	**80%**
Was the device comfortable to use?	**90%**
Was the device easy to manipulate?	**90%**
Did you find any elements of the device cumbersome?	**25%**
Did you find any features or elements of the device unnecessary?	**0%**
Did you find the device features to be intuitive?	**80%**
**Device Ultrasound Related**	
Was the ultrasound feed stable (no significant lags, freezes)?	**85%**
Did you have difficulty orienting yourself to the ultrasound?	**45%**
Was your insertion target easy to identify on the ultrasound?	**65%**
On your first attempt, did the needle insert into the location you expected?	**75%**
**Device Operation Related**	
Did you feel confident using this device?	**80%**
Was the device easy to use?	**95%**
Did you find the device helpful?	**90%**
Did you feel safe using this device?	**95%**
Did you find use of the device frustrating?	**20%**
Did you find yourself making mistakes when using the device?	**30%**
Did the device malfunction in any way during testing?	**0%**
Did this device perform as you expected?	**100%**
If you made multiple insertions, was usage of the device consistent?	**40%**
Do you think this device could be used by someone without central line placement experience?	**65%**
Do you feel satisfied using the device?	**85%**
Overall, did you like using the device?	**85%**
Would you use this device to obtain central line access on a patient?	**80%**
Did you experience any side effects or injuries while using the device?	**0%**
Do you see this device as a clinical tool?	**80%**

Do you see this device as an educational tool?	**85%**
Is this device feasible for use in remote, austere environments?	**85%**
